# An Nrf2-NF-κB Crosstalk Controls Hepatocyte Proliferation in the Normal and Injured Liver

**DOI:** 10.1016/j.jcmgh.2025.101480

**Published:** 2025-02-17

**Authors:** Andrii Kuklin, Coenraad Frederik Slabber, Luigi Tortola, Chan Lap Kwan, Gerhard Liebisch, Vangelis Kondylis, Florian Mair, Manfred Kopf, Achim Weber, Sabine Werner

**Affiliations:** 1 Institute of Molecular Health Sciences, Department of Biology, ETH Zurich, Zurich, Switzerland; 2 Department of Pathology and Molecular Pathology, University of Zurich and University Hospital Zurich, Zurich, Switzerland; 3 Institute of Molecular Cancer Research, University of Zurich, Zurich, Switzerland; 4 Institute of Clinical Chemistry and Laboratory Medicine, University of Regensburg, Regensburg, Germany; 5 Department of Gastroenterology, Hepatology and Infectious Diseases, University of Düsseldorf, Düsseldorf, Germany

**Keywords:** Nrf2, p65, Hepatocyte Proliferation, Liver Regeneration

## Abstract

**Background & Aims:**

The liver has remarkable regenerative and detoxification capacities, which require the Nrf2 and NF-κB transcription factors. Although their individual functions in hepatocytes are well characterized, knowledge about their crosstalk in the adult liver is limited.

**Methods:**

We performed AAV8-Cre inducible, hepatocyte-specific knockout of *Nrf2*, the NF-κB subunit *p65*, or both genes to determine the individual and combined roles of these transcription factors in the intact liver of male adult mice and after acute CCl_4_ injury. Mice were characterized using histologic and immunohistochemical stainings, serum and liver bile acid analysis, flow cytometry, and RNA sequencing. To distinguish between cell-autonomous and non-cell-autonomous mechanisms, we generated and analyzed knockout and knockdown AML12 liver cells. Clodronate liposome-mediated macrophage depletion was used to determine the role of these immune cells in hepatocyte proliferation after CCl_4_ injection.

**Results:**

Loss of *p65* alone or *p65* in combination with *Nrf2* caused spontaneous liver inflammation and necrosis. Gene expression profiling identified individual and common target genes of both transcription factors, including genes involved in the control of cell proliferation. Consistent with the expression of these genes, hepatocyte proliferation was reduced by Nrf2 deficiency under homeostatic conditions and after CCl_4_ injury, which was rescued by additional loss of p65. The increased hepatocyte proliferation in the double-knockout mice was non-cell-autonomous and correlated with macrophage accumulation in the liver. Depletion of macrophages in these mice suppressed hepatocyte proliferation after CCl_4_ treatment.

**Conclusions:**

These results reveal a crosstalk between Nrf2 and p65 in the control of hepatocyte proliferation and point to a key role of macrophages in this effect.

## Graphical abstract

**Figure:**
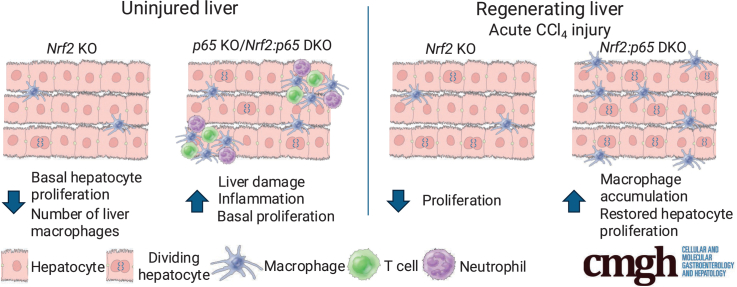

**Summary**

We identified a crosstalk between the Nrf2 and NF-κB transcription factors in hepatocytes of the normal and regenerating liver. Nrf2 deficiency suppressed hepatocyte proliferation, which was rescued by additional deficiency of p65 in a macrophage-dependent manner.

This article has an accompanying editorial.

The liver is an organ with high metabolic activity and is responsible for the detoxification of a wide variety of endogenous and exogenous compounds.^1^^,^^2^ Because of its unique place in the body and blood supply, it is frequently subjected to insults of bacterial, viral, or chemical origin, which result in increased production of reactive oxygen species (ROS).^3^ Although powerful cytoprotective and detoxification mechanisms of the liver provide effective ROS clearance, excessive accumulation of ROS often contributes to impaired liver regeneration and chronic liver damage, which may cause liver fibrosis, cirrhosis, and liver cancer, which are frequently lethal.^3^

Nuclear factor erythroid 2-related factor 2 (NRF2), which belongs to the cap’n’collar family of basic leucine zipper transcription factors, efficiently protects from oxidative and electrophilic stress in many cell types, including hepatocytes.^4^, ^5^, ^6^, ^7^ This is particularly relevant in the presence of high levels of ROS or electrophiles, which lead to stabilization of NRF2 and its accumulation in the nucleus. NRF2 regulates a wide variety of antioxidant and cytoprotective genes through binding to antioxidant response elements^8^ in their promoters and/or enhancers.

We and others showed cytoprotective functions of Nrf2 in the murine liver after different types of injury, including partial hepatectomy.^4^^,^^5^^,^^9^^,^^10^ Liver regeneration was delayed in *Nrf2* knockout (KO) mice, which was caused at least in part by impaired insulin/insulin-like growth factor receptor and Notch signaling, resulting in decreased proliferation and increased apoptosis of hepatocytes.^5^^,^^11^ Nevertheless, the liver finally regenerated, indicating compensatory functions exerted by other genes/proteins. A top candidate to fulfil this function is the nuclear factor kappa-light-chain-enhancer of activated B cells (NF-κB), which was hyperactivated after partial hepatectomy in *Nrf2* KO mice.^5^

NF-κB is a homodimeric or heterodimeric transcription factor consisting of the subunits p65 (RelA), RelB, c-Rel, p50, or p52, with the most abundant form being a heterodimer of p50 and p65.^12^ NF-κB can exert proinflammatory or anti-inflammatory activities depending on the cell type and context. In addition, it is a major player in the regulation of programmed cell death.^12^^,^^13^ This activity is highly relevant in the liver, where NF-κB protected hepatocytes against tumor necrosis factor-α-induced apoptosis.^14^, ^15^, ^16^ NF-κB is also an important player in the oxidative stress response.^17^ ROS can regulate NF-κB at different levels, resulting in its activation or inhibition in a context- and cell type–specific manner.^17^^,^^18^

Various studies showed a crosstalk between Nrf2 and NF-κB, whereby a reciprocal negative regulation was observed in most studies.^5^^,^^19^, ^20^, ^21^, ^22^, ^23^, ^24^, ^25^, ^26^, ^27^ However, we previously discovered a synergistic cytoprotective activity of both proteins in the liver.^28^ Mice lacking Nrf2 in all cells and the NF-κB subunit p65 in hepatocytes spontaneously developed progressive liver inflammation, necrosis, and fibrosis, whereas single KO mice exhibited only a minor phenotype.^28^ Moreover, 50% of aged *Nrf2:p65* double KO (DKO) female mice developed tumors resembling inflammatory hepatocellular adenomas in humans.^28^ However, it was unclear if the loss of Nrf2 in nonparenchymal cells (NPCs) of these mice contributes to the phenotype. In addition, the consequences of acute deficiency of both transcription factors in hepatocytes of adult mice and the relevant genes and pathways that they control in this cell type remained unknown.

In this study, we addressed these questions and determined the crosstalk between Nrf2 and p65 in hepatocytes at the molecular level and its relevance for hepatocytes in adult mice during homeostasis and regeneration. We demonstrate that both transcription factors collaborate in hepatocytes to control inflammation and hepatocyte proliferation.

## Results

### NF-κB-p65 and Nrf2 in Hepatocytes Protect from Liver Inflammation and Fibrosis

**Figure 1:**
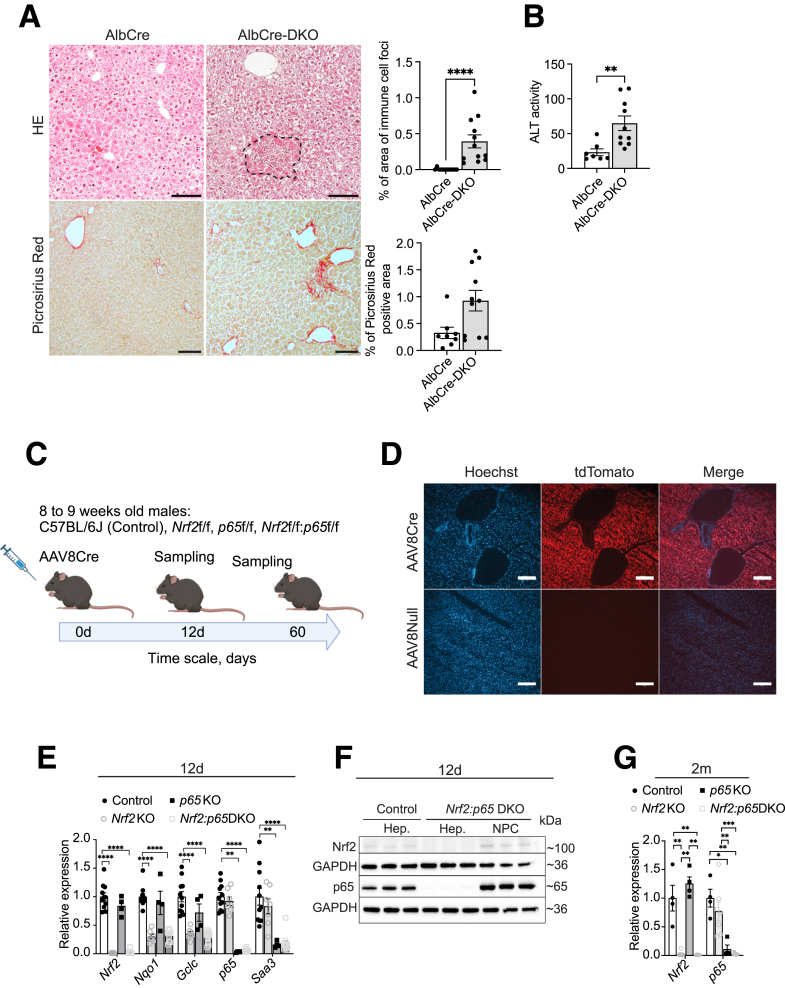
**Hepatocyte-specific deletion of *Nrf2* and *p65* leads to liver damage and fibrosis in AlbCre-*Nrf2:p65* DKO mice.** (*A*) Hematoxylin and eosin (H&E) or picrosirius red staining of liver sections from AlbCre (control) and AlbCre-*Nrf2:p65* DKO mice (AlbCre-DKO) and quantification of the stained area (N = 8–12 mice/group). (*B*) Serum activity of ALT in AlbCre and AlbCre-DKO mice (N = 7–10 mice/group). (*C*) Scheme of the experiments to induce an AAV8Cre-inducible knockout in hepatocytes. (*D*) Fluorescence microscopy analysis of liver sections from Rosa26-tdTomato mice, 12 days after AAV8Cre or AAV8Null (control virus without Cre) injection. (*E*) RT-qPCR analysis using RNA from freshly isolated hepatocytes of *Nrf2* and *p65* single KO and DKO mice at 12 days after AAVCre injection for *Nrf2*, *p65*, *Nqo1*, *Gclc*, and *Saa3* relative to *Gapdh* (N = 4–10 mice/group). (*F*) Western blot analysis using lysates from freshly isolated hepatocytes and NPCs of mice 12 days after AAV8Cre injection for Nrf2, p65, and GAPDH (glyceraldehyde 3-phosphate dehydrogenase) (loading control). (*G*) RT-qPCR analysis for *Nrf2* and *p65*, relative to *Gapdh*, using RNA from freshly isolated hepatocytes of *Nrf2* or *p65* KO or DKO mice 2 months after AAV8Cre injection (N = 4–8 mice/group). Scale bars: 100 μm (*A*), 200 μm (*B*). Bar graphs show mean and standard error of the mean. ∗*P* ≤ .05, ∗∗*P* ≤ .01, ∗∗∗*P* ≤ .001, ∗∗∗∗*P* ≤ .0001 (Mann-Whitney *U* test).

Mice with a global KO of *Nrf2* and a hepatocyte-specific KO of *p65* in hepatocytes develop spontaneous liver inflammation and fibrosis.^28^ To determine the specific contribution of Nrf2 in hepatocytes to this phenotype, we generated mice lacking both genes specifically in hepatocytes, by breeding mice expressing Cre recombinase under control of the albumin promoter (AlbCre mice) with mice carrying floxed alleles for both *Nrf2* and *p65.* Histologic analysis revealed spontaneous liver inflammation in AlbCre-*Nrf2:p65* DKO mice (AlbCre-DKO) (Figure 1*A*). They also exhibited a significant increase in the serum activity of alanine aminotransferase (ALT) and a mild, but nonsignificant increase in collagen deposition in the liver (Figure 1*A* and *B*). These results demonstrate cell-autonomous roles of Nrf2 and p65 in hepatocytes in the control of postnatal liver development and homeostasis.

### AAV8Cre Efficiently Deletes *Nrf2* and *p65* in Mouse Hepatocytes In Vivo

To determine the consequences of acute hepatocyte-specific ablation of *Nrf2*, *p65*, or both genes in adult male mice, we used Adeno-Associated Virus, serotype 8, with the *Cre* recombinase gene integrated in its genome (AAV8Cre) (Figure 1*C*). AAV8 has a strong tropism for hepatocytes.^29^ When combined with the hepatocyte-specific thyroxine-binding globulin promoter that controls *Cre* expression, it induces a hepatocyte-specific KO of *Nrf2* and *p65* in mice carrying the respective floxed alleles. All studies were performed with male mice because of the higher transduction efficiency of adeno-associated virus in male compared with female mice.^30^^,^^31^ To validate the protocol, we injected AAV8Cre into Rosa26-tdTomato reporter mice, in which expression of tdTomato is dependent on the presence of Cre. Fluorescence microscopy revealed that AAV8Cre efficiently delivered Cre throughout the liver (Figure 1*D*).

A single injection of AAV8Cre into mice with floxed *Nrf2* and/or *p65* alleles induced an efficient and long-lasting KO in hepatocytes, but not in NPCs, as demonstrated by quantitative reverse transcription polymerase chain reaction (RT-qPCR) and Western blot analysis of mouse liver at Day 12 and 2 months after virus injection (Figure 1*E-G*). Consistently, expression of the classical Nrf2 target genes *Gclc* (encoding glutamate cysteine ligase catalytic subunit) and *Nqo1* (encoding NAD(P)H quinone dehydrogenase 1) and the p65 target gene *Saa3* (encoding serum amyloid A3) was strongly downregulated in isolated hepatocytes of the respective single KO and of DKO mice (Figure 1*E*).

### Acute p65 Deficiency Induces Spontaneous Liver Damage and Focal Inflammation

**Figure 2:**
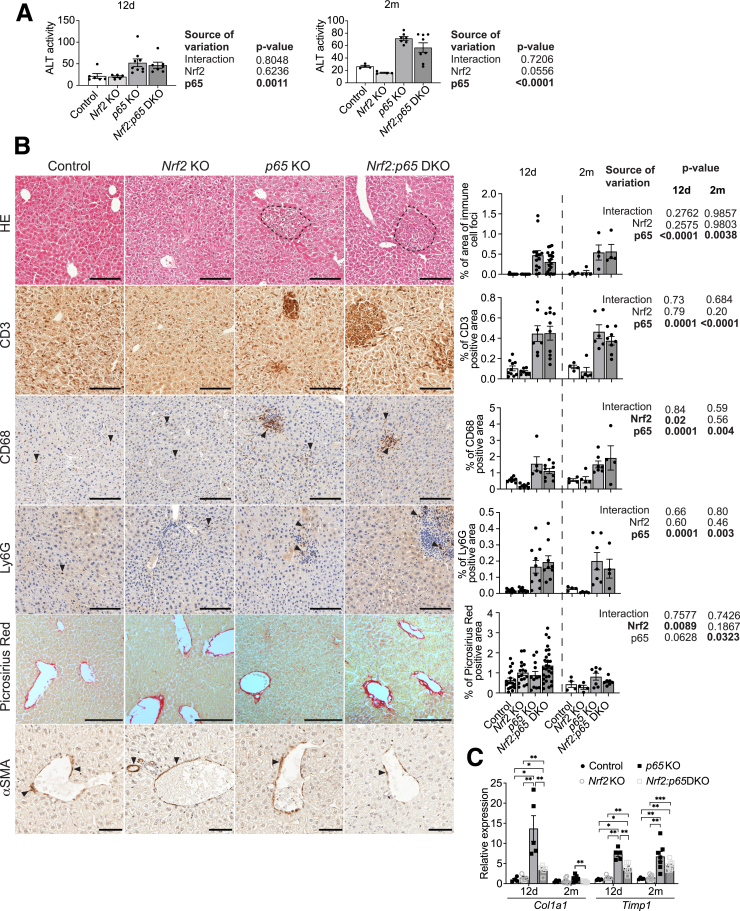
**Liver damage and inflammation after inducible deletion of *p65* in hepatocytes.** (*A*) Serum activity of ALT 12 days (12d) (N = 5–9 mice/group) and 2 months (2m) (N = 4–8 mice/group) after knockout induction. (*B*) Representative H&E or immunohistochemistry stainings of liver sections at 12 days (12d) or 2 months (2m) (only quantification) post AAV8Cre injection and quantification of the data. From *top* to *bottom*: H&E staining and quantification of the area covered by immune cell foci (N [12d] = 10–20 mice/group; N [2m] = 4 mice/group); immunohistochemical stainings for the T-cell marker CD3 (N [12d] = 7–10 mice/group; N [2m] = 4–8 mice/group); the neutrophil marker Ly6G (N [12d] = 8–10 mice/group; N [2m] = 4–7 mice/group); the macrophage marker CD68 (N [12d] = 5–9 mice/group; N [2m] = 4–7 mice/group) and quantification of the positively stained area; picrosirius red staining (N [12d] = 12–28 mice/group; N [2m] = N = 4–7 mice/group) and quantification of the stained area; representative immunohistochemical stainings for α-smooth muscle actin (αSMA) on liver sections 12d after AAV8Cre injection. Note the perivascular staining and the lack of stellate cell dissemination into parenchyma (N = 5–7). (*C*) RT-qPCR analysis for *Col1a1* and *Timp1*, relative to *Gapdh*, using RNA from freshly isolated NPCs of control, *Nrf2*, or *p65* KO or DKO mice 12d and 2m after AAV8Cre injection (N = 4–8 mice/group). Scale bars: 100 μm and 50 μm (αSMA). Bar graphs show mean and standard error of the mean. Statistical analysis: (*A, B*) 2-way analysis of variance (significant differences are in bold). (*C*) Mann-Whitney *U* test, ∗*P* ≤ .05, ∗∗*P* ≤ .01, ∗∗∗*P* ≤ .001.

Histologic analysis of hematoxylin and eosin–stained liver sections did not reveal major changes in the overall structure of the liver parenchyma in mice of all genotypes at Day 12 after KO induction. However, *p65* deletion caused spontaneous focal liver damage and inflammation, which was accompanied by a significant increase in ALT activity (Figure 2*A* and *B*). The extent of inflammation and liver damage was not aggravated by additional loss of *Nrf2* (Figure 2*A* and *B*). Immunohistochemical staining revealed accumulation of CD3-positive T cells, Ly6G-positive neutrophils, and CD68-positive macrophages within the inflammatory cell foci in the liver of *p65* KO and *Nrf2:p65* DKO mice (Figure 2*B*). Macrophage abundance was mildly reduced in *Nrf2* KO, but increased in DKO mice (Figure 2*B*). Picrosirius red staining showed only a very mild increase in perivascular collagen deposition in *Nrf2* KO and DKO mice (Figure 2*B*). Similarly, α-smooth muscle actin showed the expected perivascular localization, but there were no signs of activated stellate cells migrating into the parenchyma that would reflect induction of considerable liver fibrosis (Figure 2*B*).

Analysis of the mice 2 months post AAV8Cre injection revealed that liver damage and inflammation were long-lasting (Figure 2*A* and *B*). The activity of ALT in *p65* KO and DKO mice was even higher than at the earlier time point (Figure 2*A*), suggesting that the phenotype was persistent and even mildly progressive. However, there was no further increase in the deposition of collagen (Figure 2*B*), suggesting that the relatively low level of inflammation compared with classical fibrosis models, such as chronic CCl_4_ treatment,^32^ is not sufficient to induce a strong fibrotic phenotype. Consistent with this assumption, expression of *Col1a1*, which encodes the major type of collagen, was only transiently increased in NPCs of *p65* KO mice, and declined again at later stages. Expression of *Timp1*, which encodes a matrix metalloproteinase inhibitor, was increased in *p65* KO and DKO mice at both time points, suggesting impaired resolution of fibrosis (Figure 2*C*). Collectively, these data demonstrate that loss of *p65* in hepatocytes of adult mice rapidly induces liver damage and inflammation, but only mild fibrosis, and that additional Nrf2 deficiency does not aggravate the inflammatory phenotype in this setting.

**Figure 3:**
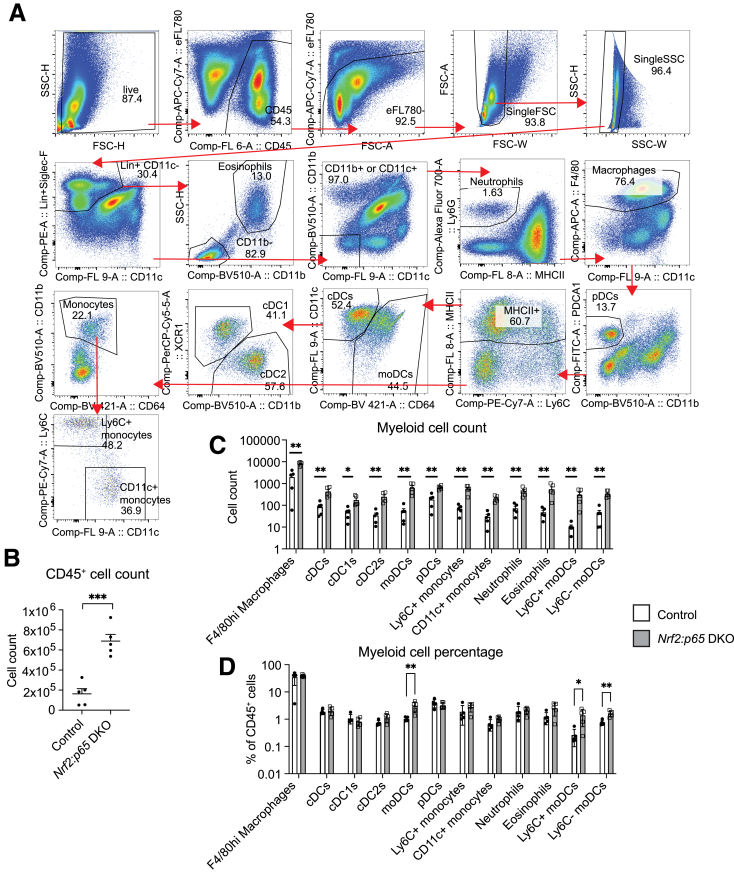
**Immune cell accumulation in *Nrf2:p65* DKO mice 12 days after knockout induction.** (*A*) Gating strategy. (*B, C*) Counts of CD45^+^ (*B*) and myeloid cells (*C*). (*D*) Relative abundance of myeloid cells among all CD45^+^ cells. Bar graphs show mean and standard error of the mean. ∗*P* ≤ .05, ∗∗*P* ≤ .01, ∗∗∗*P* ≤ .001 (unpaired t-test). N = 5 mice/group.

To further characterize the myeloid cell composition in the liver, we performed flow cytometry analysis of NPCs 12 days after KO induction (see gating strategy in Figure 3*A*), focusing on DKO versus control mice. There was a significantly higher number of CD45^+^ immune cells in the DKO mice (Figure 3*B*), which is consistent with the immunohistochemistry data. The numbers of all analyzed myeloid cell populations (monocytes, macrophages, dendritic cells, neutrophils, and eosinophils) were increased (Figure 3*C*), but the percentage of the different immune cell populations among all CD45^+^ cells was not considerably altered (Figure 3*D*).

### Combined Loss of Nrf2 and p65 Promotes Hepatocyte Proliferation in the Liver

**Figure 4:**
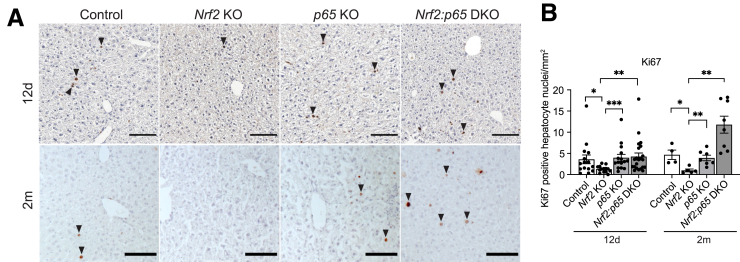
**Combined loss of Nrf2 and p65 promotes hepatocyte proliferation in the liver.** (*A*) Ki67 immunohistochemical staining of liver sections and quantification (*B*) of Ki67^+^ hepatocyte nuclei. 12d, 2m: 12 days (N = 14–21 mice/group) or 2 months (N = 4–8 mice/group) after AAV8Cre injection, respectively. Bar graphs show mean and standard error of the mean. ∗*P* ≤ .05, ∗∗*P* ≤ .01, ∗∗∗*P* ≤ .001 (unpaired t-test).

To determine if the phenotype in the different mutant mice is accompanied by altered hepatocyte proliferation, we analyzed liver sections for the proliferation marker Ki67. Twelve days after KO induction, Nrf2-deficient hepatocytes displayed significantly decreased proliferation compared with control mice, which was rescued by additional deletion of *p65* (Figure 4*A* and *B*). This phenotype was even more pronounced 2 months after induction of the KO. At this time point, the additional KO of *p65* even led to significantly increased cell proliferation compared with *p65* single KO or control mice (Figure 4*A* and *B*). Of note, the extent of liver damage was similar in *p65* KO and DKO mice, suggesting that the increased proliferation in the latter is not a compensatory response to more severe injury (Figures 2*A* and *B*).

### Gene Expression Profiling Reveals a Crosstalk Between Nrf2 and p65 in the Control of Hepatocyte Proliferation

**Figure 5:**
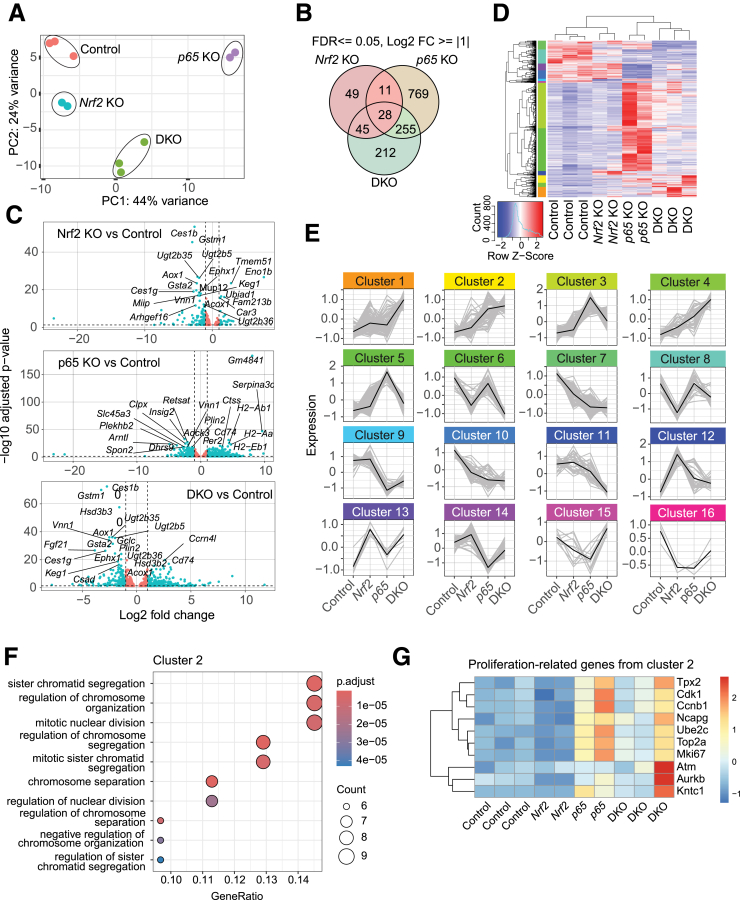
**RNA sequencing analysis reveals a crosstalk between Nrf2 and p65 in the control of hepatocyte proliferation.** (*A*) Principal component analysis of RNA sequencing data (N = 2–3 mice/group). (*B*) Venn diagram showing overlap between genes differentially expressed in freshly isolated hepatocytes from *Nrf2* and *p65* KO and DKO mice compared with hepatocytes from control mice. (*C*) Volcano plots of significantly DEGs in *Nrf2* KO, *p65* KO, and DKO hepatocytes versus control hepatocytes. Twenty highly regulated genes in each comparison are indicated. (*D*) Heatmap of the hierarchical clustering analysis of all DEGs (FDR ≤0.05) across genotypes, which were above the threshold (log_2_ fold change ≥1 or ≤-1). (*E*) Patterns of gene expression of DEGs across genotypes, allowing association of these genes with 16 clusters (Figure 7*D*). (*F*) Biologic functions of genes from cluster 2. (*G*) Heatmap showing expression of proliferation-relevant genes from cluster 2.

To gain insight into the mechanisms underlying the proliferation phenotype in the mutant mice, we performed RNA sequencing of freshly isolated hepatocytes from mice of all 4 genotypes 12 days after KO induction. Principal component analysis showed a clear clustering of the genotypes (Figure 5*A*). The single deletion of *Nrf2* or *p65* resulted in differential expression of 133 or 1063 genes, respectively, whereas their combined loss changed the expression of 540 genes compared with control subjects (false discovery rate [FDR] ≤0.05 and log_2_ fold change ≥1 or ≤-1) (Figure 5*B*, Supplementary Tables 1–3). Most of the differentially expressed genes (DEGs) in *Nrf2* KO hepatocytes were downregulated (101 of 133), confirming Nrf2 as a primarily positive regulator of gene expression. The top most significantly (lowest FDR) regulated genes in the Nrf2 dataset were related to Nrf2`s canonical function in detoxification (*Gstm1*, *Gsta2*, *Utg2b35*, *Utg2b5*, *Ugt2b36*) or involved in fatty acid metabolism (*Ces1b* and *Ces1g*) (Figure 5*C*). Among the most significantly regulated genes in *p65* KO hepatocytes were genes involved in antigen presentation (*H2-Ab1*, *H2-Aa*, *H2-Eb1*) or metabolism of retinol (*Retsat*) or lipids (*Clpx*). Expression of genes with a function in circadian regulation (*Arntl*, *Per2*) was also affected, although all mice were injected and sacrificed in the morning (Figure 5*C*). Genes encoding detoxification enzymes (*Gstm1*, *Gsta2*, *Gclc*, *Ugt2b35*, *Ephx1*), regulators of lipid metabolism (*Ces1b*, *Ces1g*, *Fgf21*), and antigen processing (*Cd74*) were the most significantly regulated genes in DKO hepatocytes (Figure 5*C*).

Next, we clustered all DEGs that showed significant regulation and determined distinct coexpression patterns among the regulated genes across all genotypes. This resulted in the identification of 16 distinct clusters (Figure 5*D* and *E*). Clusters 1 and 4 include genes with elevated expression in DKO mice. They encode proteins involved in oxygen transport, actin-based cell membrane projection and glycosaminoglycan binding, and 9 members of the Major Urinary Protein family (Supplementary Tables 4 and 5). Major urinary proteins are polymorphic proteins involved in the control of pheromone transport, regulation of behavior, and nutrient metabolism.^33^ In spite of their interesting regulation, we did not further analyze them, because they do not have orthologs in the human genome. Clusters 3 and 5 include genes that were upregulated in *p65* KO mice (Figure 5*D* and *E*). Many of them are relevant to inflammation (Supplementary Tables 4 and 5), which is consistent with the inflammatory phenotype in these mice (Figure 2*A* and *B*). Expression of most of these genes was lower in DKO hepatocytes but still higher than in hepatocytes from control mice (Figure 5*E*). Although this suggests that additional *Nrf2* deletion ameliorates liver inflammation; the altered expression did not result in major differences in immune cells between *p65* KO and DKO mice (Figure 2*B*).

Clusters 6 and 8 include genes with reduced expression in *Nrf2* KO and DKO hepatocytes compared with control and *p65* KO hepatocytes. They include classical Nrf2 target genes involved in detoxification and lipid metabolism (Figure 5*E*, Supplementary Tables 4 and 5).

Cluster 13 includes genes with an opposite expression profile compared with clusters 6 and 8, indicating that Nrf2 represses their expression (Figure 5*E*). Among them are the genes for calcium/calmodulin-dependent protein kinase II beta (*Camk2b*), which is involved in the suppression of insulin signaling,^34^ protein phosphatase 1, regulatory subunit 3G (*Ppp1r3g*), known to regulate glycogen synthesis,^35^ and the tumor suppressor Rap1 GTPase-activating protein (*Rap1gap*) (Supplementary Tables 4 and 5). Genes from cluster 12 were also upregulated in response to *Nrf2* deletion, but their expression was unchanged in DKO mice (Figure 5*E*). Among them are enzymes with documented roles in the pathogenesis of hepatocellular carcinoma (*Sult1c2* and *St6galnac4*),^36^ or alcohol-induced steatosis (*Pde4*).^37^

Clusters 7 and 10 include genes with reduced expression in *Nrf2* single KO mice and a further reduction in *p65* KO and DKO mice (Figure 5*E*). Their products are involved in fatty acid metabolism and include acyl-CoA thioesterase family members (*Acot1*, *Acot2*) and members of the cytochrome P-450 monooxygenase family (*Cyp4a31*, *Cyp4a14*, *Cyp4a32*, and *Cyp4a10*) (Supplementary Tables 4 and 5).

The basal expression of genes from clusters 9 and 14 was dependent on p65; however, additional deletion of *Nrf2* in DKO mice partially rescued their decreased expression (Figure 5*E*). Among them are genes encoding tight junction components (*Cldn1*, *Tjp1*, *Tjp2*); enzymes involved in very-long-chain fatty acid metabolism (*Elovl3*, *Elovl5*); components of the Map kinase signaling pathway (*Map3k13*, *Map4k4*); and the *Ggct* gene, which is involved in glutathione catabolism (Supplementary Tables 4 and 5).

Cluster 2 included several genes related to cell proliferation (Figure 5*E-G*, Supplementary Tables 4 and 5). They encode cell cycle regulators (*Cdk1*, *Ccnb1*, *Aurkb*, *Ube2c*) and proteins involved in chromosome segregation during mitosis (*Ncapg*, *Mki67*, *Top2a*, *Tpx2*, *Kntc1*). Their differential expression correlates with the alterations in cell proliferation identified by Ki67 staining (Figure 4*A* and *B*). These findings point to an interplay between Nrf2 and p65 in the control of hepatocyte proliferation.

**Figure 6:**
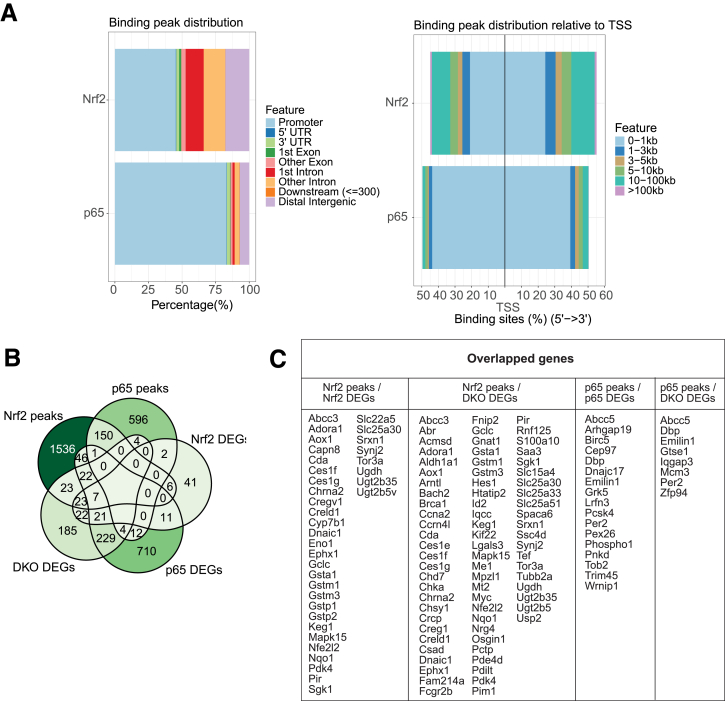
**Analysis of Nrf2 and p65 ChIP-seq data.** (*A*) Distribution of Nrf2 and p65 binding peaks within different DNA elements and relative to the transcription start site (TSS). (*B*) Venn diagram showing the overlap between DEGs (*Nrf2*-, *p65*-, and DKO vs *ctrl*) and genes that contain binding peaks of Nrf2 and p65. (*C*) The list of overlapping genes from *B*.

To determine if some of the DEGs are directly regulated by Nrf2 and p65 in the liver, we analyzed published chromatin immunoprecipitation sequencing (ChIP-seq) data that show DNA binding of Nrf2 or p65 in the liver of male C57BL/6 mice (GSE109865,^38^ GSE117488,^39^ both taken from Gene Expression Omnibus database, https://www.ncbi.nlm.nih.gov/geo/). Nrf2 binding sites were broadly distributed and present in promoters and distal enhancers, whereas p65 bound predominantly in proximal promoter regions (Figure 6*A*). There was a clear overlap between DEGs from *Nrf2* KO and DKO hepatocytes with Nrf2 ChIP-seq data, showing that 36 genes that were regulated in *Nrf2* KO hepatocytes and 75 genes that were regulated in DKO hepatocytes are direct Nrf2 targets (Figure 6*B* and *C*). Among them are genes encoding key regulators of redox homeostasis and compound detoxification (eg, *Gstp1*, *Gstp2*, *Srxn1*, *Gstm3*, *Gstm1*, *Nqo1*, and *Gclc*) (Figure 6*B* and *C*). Only 17 or 8 genes that were differentially expressed in *p65* KO or DKO hepatocytes, respectively, overlapped with ChIP-seq binding peaks (Figure 6*B* and *C*). We did not identify binding peaks in the promoters of proliferation-related genes from cluster 2, suggesting that they are not directly regulated by Nrf2 or p65.

### Loss of *Nrf2* Reduces Hepatocyte Proliferation in the Injured Liver, which is Rescued by Additional *p65* Deficiency

**Figure 7:**
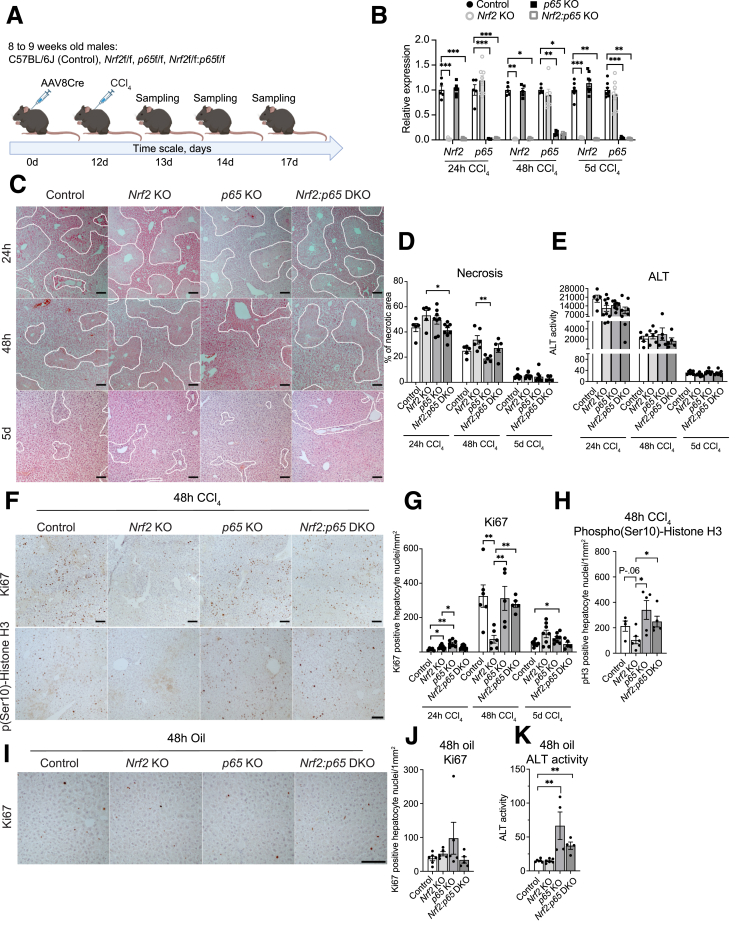
**Deletion of *p65* rescues the proliferation defect of Nrf2-deficient hepatocytes in the regenerating liver.** (*A*) Scheme of the experimental setup, including AAV8Cre injection and CCl_4_ treatment. (*B*) RT-qPCR for *Nrf2* and *p65* relative to *Gapdh* using RNA from freshly isolated hepatocytes of control, *Nrf2*, and *p65* KO and DKO mice, 24 hours (N = 5–8 mice/group), 48 hours (N = 4–6 mice/group), and 5 days (N = 5–8 mice/group) after CCl_4_ treatment. (*C*) H&E staining of liver sections from control, *Nrf2*, and *p65* KO and DKO mice at different time points post CCl_4_ injection (N = 5–8 mice/group). (*D*) Quantification of the necrotic area (encircled with a *white line* in *C*). (*E*) Serum activity of ALT at different time points post CCl_4_ injection (N = 5–9 mice/group). (*F*) Ki67 and phospho-(Ser10)-histone H3 immunohistochemistry staining of liver sections and (*G, H*) quantification (N = 5–8 mice/group and N = 4–6 mice/group respectively). (*I*) Representative immunohistochemical staining of liver sections for Ki67, 48 hours after olive oil injection, and quantification (*J*) of the stained cells (N = 5–6 mice/group). (*K*) Serum activity of ALT in mice treated with olive oil for 48 hours (N = 4–6 mice/group). Scale bars: 100 μm. Bar graphs show mean and standard error of the mean. ∗*P* ≤ .05, ∗∗*P* ≤ .01, ∗∗∗*P* ≤ .001 (Mann-Whitney *U* test).

We next determined if the differences in proliferation observed in the noninjured liver of mice with different genotypes are retained or even exacerbated after liver injury, when the need for proliferation is increased. Therefore, we injected the mice with the hepatotoxin carbon tetrachloride (CCl_4_), which causes hepatocyte death with compensatory hyperproliferation after a single application. Mice were sacrificed and analyzed 24 hours, 48 hours, and 5 days after CCl_4_ injection (Figure 7*A*). To monitor a possible overgrowth of hepatocytes, which had not been infected with AAV8-Cre and/or escaped recombination, we monitored the KO efficiency in freshly isolated hepatocytes from the regenerating liver and confirmed that expression of both *Nrf2* and *p65* was strongly downregulated in single KO and DKO mice (Figure 7*B*). There was a mildly larger necrotic area in *Nrf2* KO mice compared with control mice at 48 hours after CCl_4_ injection (Figure 7*C* and *D*). Serum activity of ALT was comparable in mice of all genotypes at 24 hours and 48 hours post CCl_4_ injection (Figure 7*E*). Ki67 staining confirmed the data obtained with uninjured liver and showed a strong and significant reduction of Ki67-positive hepatocytes in *Nrf2* KO mice and a rescue of this phenotype in DKO mice at 48 hours after CCl_4_ injection, the time point that corresponds to the maximum proliferation rate of hepatocytes (Figure 7*F* and *G*). A similar result was obtained with another proliferation marker, phosphorylated histone H3 (Figure 7*F* and *H*). No difference in proliferation was seen in the oil-treated control mice, and the serum activity of ALT in these mice was comparable with untreated mice (Figure 7*I-K*).

Together, these results demonstrate that Nrf2 is important for hepatocyte proliferation after CCl_4_ injury and that additional loss of p65 rescues the proliferation defect caused by the loss of Nrf2 in these cells.

### p65 Deficiency Rescues the Proliferation Defect of Nrf2-Deficient Hepatocytes Via Non-Cell-Autonomous Mechanisms

**Figure 8:**
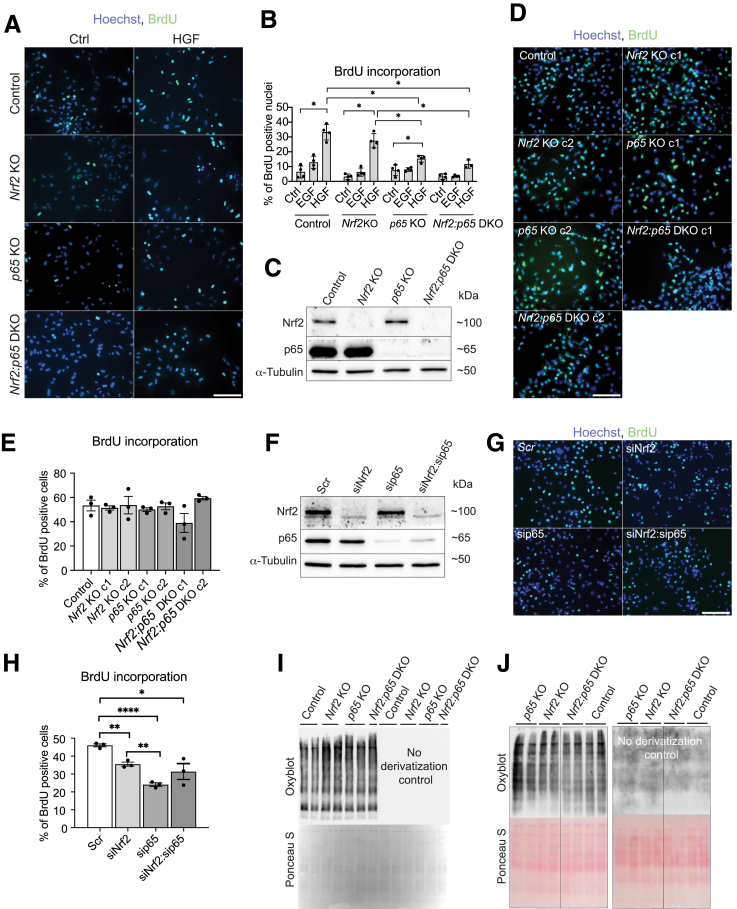
**Reduced hepatocyte proliferation in *Nrf2* KO mice and its rescue by *p65* deletion is mediated by non-cell-autonomous mechanisms.** (*A, B*) Representative BrdU immunofluorescence stainings (*A*) and quantification (*B*) of BrdU^+^ cultured primary hepatocytes from control, *Nrf2*, and *p65* KO and DKO mice treated with 20 ng/mL of epidermal growth factor (EGF), hepatocyte growth factor (HGF), or vehicle for 40 hours (N = 3–4 cell culture wells/group). (*C*) Western blotting for Nrf2, p65, and α-tubulin (loading control) using lysates from control, *Nrf2*, and *p65* KO and DKO AML12 cell lines. (*D, E*) Representative immunofluorescence stainings (*D*) and quantification (*E*) of BrdU^+^ AML12 cells. c1, c2 denotes different clones of the same gene knockout (N = 3 cell culture wells/group). (*F*) Western blot analysis for Nrf2, p65, and α-tubulin using lysates from AML12 cells transfected with scrambled (Scr) Nrf2, p65, or Nrf2 and p65 siRNAs for 48 hours. Representative immunofluorescence stainings (*G*) and quantification of BrdU^+^ AML12 cells transfected with the different siRNAs for 48 hours (*H*) (N = 3 cell culture wells/group). Analysis of oxidized proteins in total liver from control, *Nrf2*, and *p65* KO and DKO mice 12 days after knockout induction, before (*I*) and 48 hours after CCl_4_ treatment (*J*) using oxyblot. The antibody specificity was assessed by omitting the derivatization step (*right part of the gels*). Protein loading was assessed by Ponceau S staining of the membrane (*lower panels*). Scale bars: 100 μm. Bar graphs show mean and standard error of the mean. ∗*P* ≤ .05, ∗∗*P* ≤ .01, ∗∗∗∗*P* ≤ .0001 (2-tailed t-test).

To determine if the proliferation defect of Nrf2-deficient hepatocytes and its rescue by additional loss of p65 is cell-autonomous, we analyzed the response of cultured primary hepatocytes from the mutant mice to the primary hepatocyte mitogens hepatocyte growth factor or epidermal growth factor using BrdU incorporation studies. However, the hepatocyte growth factor–mediated increase in hepatocyte proliferation was not affected by the loss of Nrf2, and the additional p65 deficiency even reduced the response (Figure 8*A* and *B*). Epidermal growth factor had no significant effect on cell proliferation, independent of the genotype (Figure 8*B*).

Next, we generated single KO and DKO for *Nrf2* and *p65* in the murine AML12 hepatocyte cell line using CRISPR/Cas9 genome editing (Figure 8*C*). There was no difference in BrdU incorporation between the different cell lines (Figure 8*D* and *E*). However, when Nrf2, p65, or both transcription factors were acutely depleted using siRNA (Figure 8*F*), we observed a mildly reduced proliferation rate of the Nrf2 knock-down cells and an even stronger reduction in p65 knock-down cells compared with AML12 cells transfected with scrambled siRNA (Figure 8*G* and *H*). Nevertheless, no rescue of the proliferation defect was observed in double knock-down cells. Collectively, these findings strongly suggest that the rescue of the proliferation defect of Nrf2-deficient hepatocytes by additional loss of p65 in vivo involves non-cell-autonomous mechanisms.

Because both Nrf2 and p65 control ROS levels in hepatocytes, and excessive ROS levels, in turn, can suppress proliferation,^5^ we determined the ROS-induced protein oxidation 12 days after KO induction and 48 hours after CCl_4_ treatment. However, there was no correlation between the amounts of oxidized proteins and the extent of cell proliferation in the intact or regenerating liver (Figures 8*I* and *J*).

### Alterations in Bile Acids and Lipid Accumulation Do Not Correlate with the Altered Hepatocyte Proliferation in *Nrf2* KO and DKO Mice

**Figure 9:**
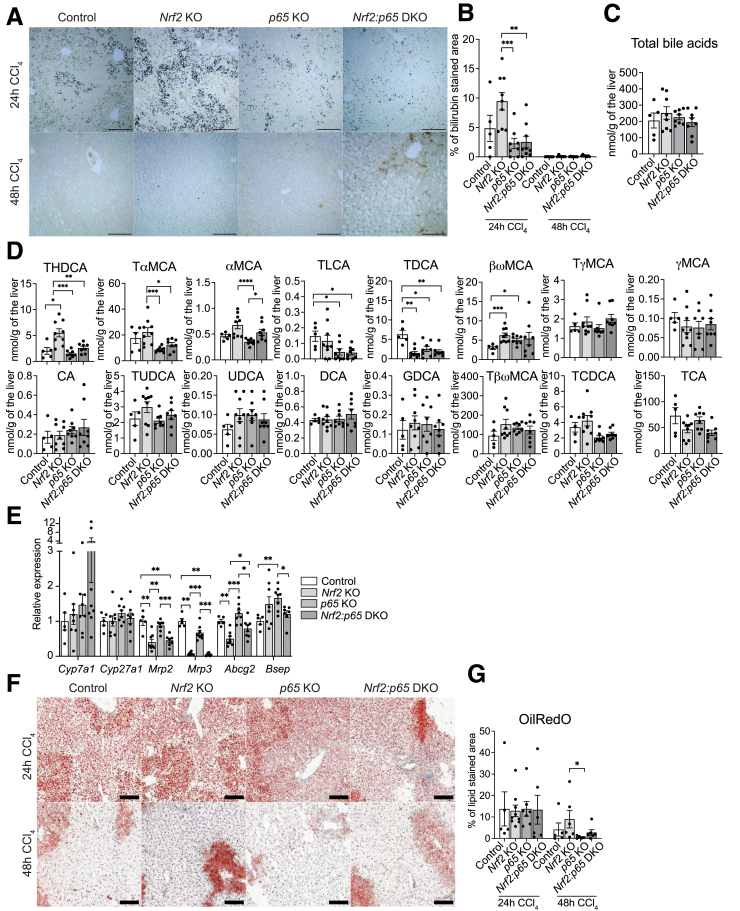
**Alterations in bile acids and lipid accumulation do not correlate with the altered hepatocyte proliferation in *Nrf2* KO and DKO mice.** (*A, B*) Representative bile pigment stainings of liver sections from control, *Nrf2*, or *p65* KO or DKO mice 24 hours and 48 hours after CCl_4_ treatment (*A*) and quantification of the stained area (*B*) (N = 5–9 mice/group). (*C*) Quantification of total bile acids (determined by mass spectrometry in the liver of control, *Nrf2*, *p65* KO, and *D*KO mice 24 hours after CCl_4_ treatment (N = 5–9 mice/group). (*D*) Mass spectrometry determination of individual bile acid concentrations in the liver of control, *Nrf2*, *p65* KO, and *D*KO mice 24 hours after CCl_4_ treatment (N = 5–9 mice/group). CA, cholic acid; DCA, deoxycholic acid; GDCA, glycodeoxycholic acid; αMCA, alpha-muricholic acid; βωMCA, combined quantification of beta/omega-muricholic acid; γMCA, gamma-muricholic acid; TαMCA, tauro-alpha-muricholic acid; TβωMCA, combined quantification of tauro-beta/omega-muricholic acid; TγMCA, tauro-gamma-muricholic acid; TCA, taurocholic acid; TCDCA, taurochenodeoxycholic acid; TDCA, taurodeoxycholic acid; THDCA, taurohyodeoxycholic acid; TUDCA, tauroursodeoxycholic acid; UDCA, ursodeoxycholic acid. (*E*) RT-qPCR for *Cyp7a1*, *Cyp27a1*, *Mrp2*, *Mrp3*, *Abcg2*, and *Bsep* relative to *Gapdh* using RNA from freshly isolated hepatocytes of control, *Nrf2*, and *p65* KO and DKO mice 24 hours after CCl_4_ treatment. N = 5–8 mice/group. (*F, G*) Representative oil red O lipid staining of liver sections from control, *Nrf2*, or *p65* KO or DKO mice 24 hours and 48 hours after CCl_4_ treatment (*F*) and quantification (*G*) of the stained area (N = 5–9 mice/group). Scale bars: 100 μm. Bar graphs show mean and standard error of the mean. ∗*P* ≤ .05, ∗∗*P* ≤ .01. ∗∗∗*P* ≤ .001, ∗∗∗∗*P* ≤ .0001 (Mann-Whitney *U* test).

Because of the important role of bile acids in the control of hepatocyte proliferation,^40^, ^41^, ^42^ we stained sections of CCl_4_-treated mice for the bile pigment bilirubin. Twenty-four hours after CCl_4_ treatment, the bilirubin-positive area was mildly increased in *Nrf2* KO mice and significantly reduced in *p65* KO and DKO mice (Figure 9*A* and *B*). However, at 48 hours, when differences in cell proliferation were particularly obvious (Figure 7*F-H*), bilirubin deposition was equally low in mice of all genotypes (Figure 9*A* and *B*). Mass spectrometry analysis of the liver tissue 24 hours after CCl_4_ injection showed no differences in total bile acid levels between genotypes (Figure 9*C*), but individual bile acids were altered (Figure 9*D*). The concentrations of taurolithocholic acid (TLCA), alpha-muricholic acid, and tauro-alpha-muricholic acid were significantly decreased in *p65* KO and DKO mice compared with control and *Nrf2* KO mice (Figure 9*D*), and taurodeoxycholic acid was decreased in mice of all genotypes. By contrast, beta-omega-muricholic acid levels were significantly increased in *Nrf2* KO and *p65* KO mice, and taurohyodeoxycholic acid, a key contributor to hyperlipidemia,^43^ was increased in *Nrf2* KO mice (Figure 9*D*). Together with the Nrf2-regulated expression of genes involved in fatty acid metabolism (discussed previously), the increase in taurohyodeoxycholic acid supports previous data, demonstrating that Nrf2 protects from nonalcoholic steatohepatitis in mice.^44^^,^^45^

The differential abundance of certain bile acids in the regenerating liver of *Nrf2* KO or *Nrf2:p65* DKO mice may be a consequence of the altered expression of bile acid transporters (*Mrp2*, *Mrp3*, *Abcg2*) or of the bile acid synthesis enzyme *Cyp7a1* in hepatocytes (Figure 9*E*) or result from alterations in the microbiome.

Given the important role of lipids in the control of hepatocyte proliferation^46^^,^^47^ and the regulation of lipid metabolism genes by both Nrf2 and p65 as shown in our RNA sequencing experiment, we performed oil red O lipid staining of liver sections 24 hours and 48 hours after CCl_4_ treatment. However, we only observed a mild increase in liver lipids in *Nrf2* KO mice at the 48-hour time point (Figure 9*F* and *G*).

Altogether, despite the differences in the amount of specific bile acids and subtle changes in lipid accumulation among the mice with the 4 genotypes, there was no robust correlation with the proliferation phenotype.

### Macrophages Contribute to the Increased Hepatocyte Proliferation in DKO Mice

**Figure 10:**
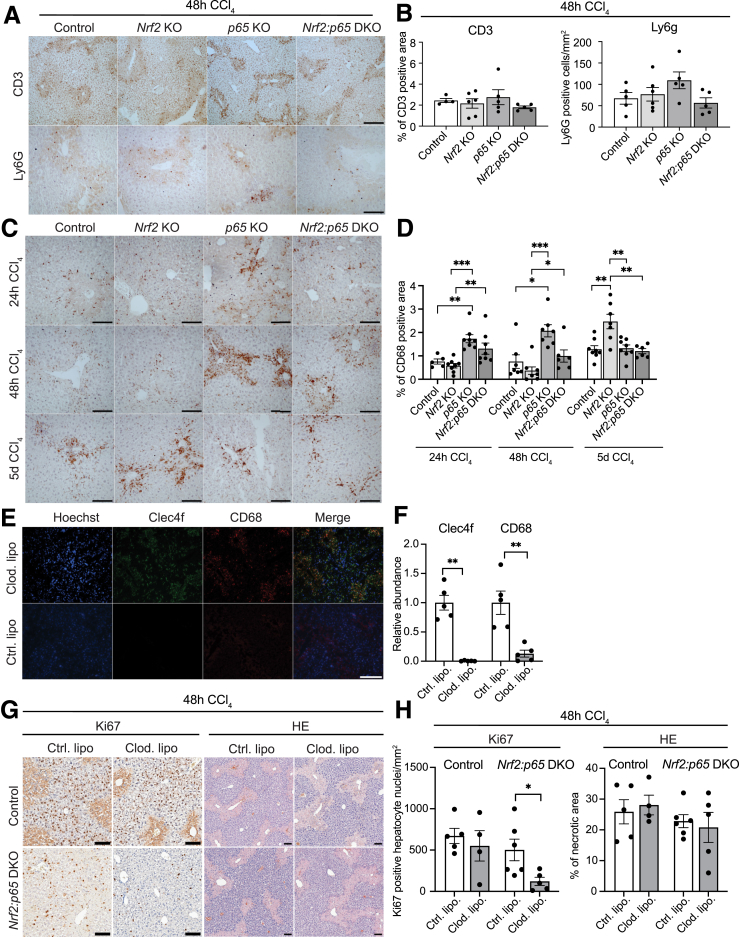
**Macrophage accumulation promotes hepatocyte proliferation in *Nrf2:p65* DKO mice following CCl**_**4**_**treatment.** (*A, B*) Representative immunohistochemical stainings of liver sections from control, *Nrf2*, and *p65* KO and DKO mice, 48 hours after CCl_4_ treatment, for CD3 and Ly6G (*A*), and quantification (*B*) of the CD3^+^ or Ly6G^+^ area (N = 4–6 mice/group). (*C, D*) Representative immunohistochemical stainings of liver sections from control, *Nrf2*, *p65* KO and DKO mice, 24 hours, 48 hours, and 5 days after CCl_4_ injection, for the macrophage marker CD68 (*C*), and quantification of the CD68^+^ area (*D*) (N = 5–8 mice/group). (*E, F*) Representative immunofluorescence stainings of liver sections from DKO mice, 48 hours after CCl_4_ injection, for the Kupffer cell marker Clec4f and the general macrophage marker CD68 (*E*), and quantification of the Clec4f^+^ and CD68^+^ area (*F*) (N = 5 mice/group). (*G, H*) Representative immunohistochemical stainings for Ki67 and H&E stainings of liver sections from control and DKO mice injected with clodronate liposomes (Clod. lipo) or control liposomes (Ctrl. lipo.) and treated with CCl_4_ for 48 hours (*G*), and quantification of Ki67^+^ positive nuclei and necrotic area (*H*) (N = 4–6 mice/group). Scale bars: 100 μm. Bar graphs show mean and standard error of the mean. ∗*P* ≤ .05, ∗∗*P* ≤ .01. ∗∗∗*P* ≤ .001 (Mann-Whitney *U* test).

Finally, we assessed if the rescue of the reduced hepatocyte proliferation in CCl_4_-treated DKO versus *Nrf2* KO mice is mediated, at least in part, by immune cells. To test this possibility, we performed immunohistochemical staining for immune cell–specific markers. On treatment with CCl_4_ for 48 hours, the abundance of T cells and neutrophils was similar in mice of all genotypes (Figure 10*A* and *B*). However, the number of macrophages was significantly increased in *p65* KO mice at 24 hours and 48 hours, and this increase was also seen in DKO mice (Figure 10*C* and *D*). On Day 5 after CCl_4_ treatment, the macrophage abundance was significantly higher in *Nrf2* single KO mice compared with other genotypes (Figure 10*C* and *D*). This corresponded to the mild increase in hepatocyte proliferation in *Nrf2* single KO mice at this time point (Figure 7*F* and *G*).

To determine if the increase in macrophages is functionally relevant, we injected control and DKO mice with CCl_4_ following macrophage depletion with clodronate liposomes. A single injection of clodronate liposomes efficiently depleted both Clec4f-positive Kupffer cells and CD68-positive liver macrophages (among which are monocyte-derived macrophages) (Figure 10*E* and *F*). This correlated with a significantly reduced proliferation rate of hepatocytes in DKO mice injected with clodronate versus control liposomes, whereas necrosis was not significantly affected (Figure 10*G* and *H*). By contrast, macrophage depletion had no significant effect on proliferation in CCl_4_-treated control mice at the 48-hour time point (Figure 10*G* and *H*). These results strongly suggest that macrophages in the *Nrf2:p65* DKO mice contribute to the rescue of the proliferation defect observed in *Nrf2* single KO mice.

## Discussion

We discovered a crosstalk between Nrf2 and p65 in hepatocytes that results in differential regulation of direct and indirect Nrf2 and/or p65 target genes in mice with single or combined deletion of these transcription factors. Of particular interest was the regulation of genes involved in cell proliferation, because their expression correlated with the observed differences in hepatocyte proliferation under homeostatic conditions and after toxin-induced injury. Hepatocyte-specific deletion of *Nrf2* in adult mice led to decreased proliferation of these cells already under homeostatic conditions and, in particular, after CCl_4_ injury. This is in line with our previous study that showed decreased hepatocyte proliferation in mice with a global and constitutive *Nrf2* KO after a single CCl_4_ injection.^4^ The severity of the proliferation defect was, however, much stronger on inducible *Nrf2* ablation in adult mice, which may have not allowed activation of compensatory mechanisms. Surprisingly, additional deletion of *p65* rescued this phenotype without significantly affecting the extent of necrotic damage.

Nrf2 may affect cell proliferation in a ROS-dependent or -independent manner. Increased ROS levels in the liver of *Nrf2* KO mice caused impaired insulin receptor/insulin-like growth factor signaling in the liver following partial hepatectomy, which suppressed hepatocyte proliferation.^5^ However, the rescue of proliferation in mice with inducible deletion of *Nrf2* by additional loss of *p65* that we observed in the present study is probably not a consequence of major alterations in ROS levels as suggested by the similar amounts of oxidized proteins in the liver of mice of all genotypes. The inhibitory effect of Nrf2 deficiency on hepatocyte proliferation is at least in part cell-autonomous, as shown by in vitro studies. We also found reduced expression of various proliferation-associated genes in freshly isolated hepatocytes of *Nrf2* single KO mice. However, ChIP-seq data showed that these genes are not directly regulated by Nrf2 in hepatocytes of noninjured liver, suggesting the involvement of indirect regulatory mechanisms. Consistent with this assumption, the number of macrophages was lower in *Nrf2* KO mice compared with mice with other genotypes under basal conditions. This may affect the availability of macrophage-derived signals that are required for the proper initiation of liver regeneration.

Our data further suggest that non-cell-autonomous mechanisms are responsible for the rescue of the in vivo proliferation phenotype in DKO mice. Of particular interest in this context is the strongly increased abundance of macrophages in DKO compared with *Nrf2* KO mice, both in the normal and in the regenerating liver. Macrophages play key roles in liver regeneration, because they produce growth factors, cytokines, chemokines, and matrix metalloproteinases that provide vital signals for hepatocytes and other liver cell types.^48^, ^49^, ^50^, ^51^, ^52^, ^53^ Consistent with these activities, macrophage depletion using clodronate liposomes caused enhanced liver necrosis and delayed hepatocyte proliferation following CCl_4_ treatment of wild-type C57BL/6J mice.^54^ However, in our study, depletion of macrophages in control mice (C57BL/6J injected with AAV8Cre) using clodronate liposomes did not significantly affect hepatocyte proliferation and necrosis following CCl_4_ treatment. This discrepancy may result from alterations in the macrophage phenotype by AAV8Cre injection or by differences in the microbiome. In *Nrf2:p65* DKO mice, macrophage depletion suppressed hepatocyte proliferation without obvious effects on necrosis. Therefore, mitogenic signals provided by macrophages in DKO mice are most likely responsible for the rescue of the proliferation phenotype that results from Nrf2 deficiency. In the future it will be of interest to further characterize the macrophage phenotype in these mice.

The reason for the macrophage accumulation in DKO mice remains unclear. RNA sequencing identified several upregulated chemokines (*Cxcl14*, *Cxcl9*, *Cxcl16*, and *Ccl6*) in *p65* KO and DKO mice. Some of them may contribute to the increased recruitment of monocytes/macrophages under basal conditions and following CCl_4_ injury, and potentially influence their phenotype. Additionally, changes in the bile acid pool could play a role. Consistent with this possibility, TLCA levels were significantly lower in *p65* KO and DKO mice compared with control and *Nrf2* KO mice. TLCA is the most potent ligand of the Tgr5 receptor that is known to suppress macrophage activity.^55^^,^^56^ Therefore, the reduced levels of TLCA in *p65* KO and DKO mice may affect the abundance and activation of macrophages.

Taken together, the results obtained in this study identify an important role of the Nrf2-p65 crosstalk in controlling hepatocyte proliferation in the normal and injured liver, which involves cell-autonomous but mainly non-cell-autonomous mechanisms. They also provide mechanistic insight into the interplay between 2 major cytoprotective transcription factors. This functional interaction not only regulates cell proliferation, but also affects ROS and compound detoxification, lipid metabolism, and antigen processing. Future investigation should identify common target genes of both transcription factors that control these processes and the mechanism of their regulation by Nrf2 and p65.

## Materials and Methods

### Mouse Models

We used 8- to 9-week-old male wild-type C57BL/6J mice, and mice with the same genetic background bearing floxed alleles for either *Nrf2*,^57^ *p65*,^58^ or both genes. Mice with floxed alleles for both *Nrf2* and *p65* were mated with transgenic mice expressing Cre recombinase under the control of the albumin promoter (AlbCre mice^59^). Mice were maintained under specific pathogen-free conditions and received food and water ad libitum. Mouse maintenance and all procedures with animals had been approved by the local veterinary authorities of Zürich, Switzerland (Kantonales Veterinäramt Zürich).

### Acute Gene KO in Hepatocytes

KO of *p65* and *Nrf2* was induced in mice carrying the respective floxed alleles by a single intraperitoneal injection of AAV8 viruses, which express Cre recombinase under the control of the thyroxine-binding globulin promoter (AAV8Cre; 9.5 x 10^10^ viral genomes in 100-μL phosphate-buffered saline [PBS]; virus provided by James M. Wilson and obtained via Addgene, Watertown, MA; #107787-AAV8). The thyroxine-binding globulin promoter is specifically active in hepatocytes. Twelve or 60 days after induction of the KO, mice were euthanized, and liver tissue was sampled. To monitor deletion efficiency of floxed alleles, we used Rosa26-tdTomato reporter mice.^60^

### CCl_4_-Induced Acute Liver Injury

Ten- to 11-week-old male mice, 12 days after KO induction, were injected intraperitoneally with a single dose of CCl_4_ (270652, Sigma-Aldrich, St. Louis, MO) (1:10 dilution of CCl_4_ in olive oil, 10 μL/g of body weight) or vehicle (olive oil) between 8 am and 12 pm. Mice were sacrificed 24 hours, 48 hours, or 5 days after injection.

### Macrophage Depletion

On Day 11 after AAV8Cre injection, mice were intravenously injected (3.5 μL/g of body weight) with clodronate-loaded or control liposomes (CP-SUV-005-005; Liposoma BV, The Netherlands). Twenty-four hours later, they were injected with CCl_4_ as described previously, and sacrificed after 48 hours.

### Isolation of Primary Hepatocytes and NPCs

Mice were anesthetized by intraperitoneal injection of Esconarkon (300 μg/g of body weight). The inferior vena cava was cannulated, and the liver was perfused for 4 minutes with prewarmed (37°C) Hank’s medium (Sigma-Aldrich) supplemented with 0.5 mM EGTA. Mouse liver cells were isolated by perfusing the liver for 5 minutes with prewarmed (37°C) digestion medium (Dulbecco’s modified eagle’s medium [DMEM]–low glucose; Sigma-Aldrich) supplemented with 1% penicillin-streptomycin (P/S; Sigma-Aldrich), 15 mM HEPES (Thermo Fisher Scientific, Waltham, MA), and 32 μg/mL Liberase (Roche, Rotkreuz, Switzerland). After digestion, cells were transferred into 20 mL of cold isolation medium (DMEM– low glucose supplemented with 1% P/S and 10% fetal bovine serum [FBS; Thermo Fisher Scientific]), filtered through a 100-μm mesh cell strainer and centrifuged at 50 x *g* for 2 minutes. Cells were then washed in 20 mL of cold isolation medium and centrifuged again. The supernatant was kept for the isolation of NPCs. The hepatocyte cell pellet was resuspended in 15 mL of cold isolation medium mixed with 10 mL of cold isotonic Percoll (1:10 10 x PBS:Percoll, GE Healthcare, Chicago, IL) and centrifuged at 50 x *g* for 10 minutes. The purified hepatocytes were washed with 25 mL of isolation medium, and either processed immediately or aliquoted, snap-frozen in liquid nitrogen, and stored at -80°C until further analyses.

For isolation of NPCs, pooled supernatants after hepatocyte precipitation were centrifuged for 5 minutes at 750 x *g*. The cell pellet was resuspended in 2.5 mL of cold PFB buffer (1 x PBS, 2% FBS, 1 mM EDTA), mixed with 2.5 mL of cold 40% isotonic OptiPrep (Stemcell Technologies, Vancouver, CA), overlayed with 2 mL of 1 x PBS and centrifuged at 1500 x *g* for 25 minutes at room temperature in a swing bucket rotor. NPCs at the Optiprep/PBS interface were collected and washed in 15 mL isolation medium, followed by centrifugation at 750 x *g* for 5 minutes. Purified NPCs were aliquoted and processed immediately for further analyses or snap-frozen in liquid nitrogen and stored at -80°C.

For flow cytometry analysis, the protocol was modified by using digestion media containing collagenase IV (600 U/mL) and DNase 1 (200 U/mL) (both from Worthington Biochemical Corporation, Lakewood, NJ) instead of Liberase.

### Collection of Liver Samples

Before proceeding to perfusion and cell isolation, the right lobe of the liver was ligated with a nylon thread, removed, separated into 3 pieces, and fixed in 4% paraformaldehyde (PFA) in PBS overnight at 4°C or in acetic ethanol (75% ethanol/25% acetic acid) overnight at 4°C. Alternatively, tissue samples were directly embedded in tissue tissue-freezing medium (Leica, Nussloch, Germany). PFA- and acetic ethanol-fixed tissue samples were dehydrated and embedded in paraffin.

### Immunohistochemistry Staining and Image Analysis

The 3.5-μm-thick sections from PFA- and acetic ethanol-fixed liver tissue were dewaxed and rehydrated. PFA-fixed sections were used for Ki67, CD3, and p-histone H3 staining. Acetic ethanol-fixed sections were used for CD68, Ly6G, and α-smooth muscle actin staining. Whenever needed, antigens were unmasked by incubating slides at 95°C in sodium citrate buffer (10 mM sodium citrate, 0.05% Tween 20, pH 6.0; for Ki67 and p-histone H3 staining) or in Tris-EDTA buffer (10 mM Tris, 1 mM EDTA, 0.05% Tween 20, pH 9.0; for CD3 staining), for 40 minutes. Sections were cooled down to room temperature, washed 2 x 5 minutes in phosphate-buffered saline with Tween 20 (PBST: 1 x PBS, 0.1% Tween 20), and unspecific binding sites were blocked for 1 hour in blocking solution (1% bovine serum albumin [BSA], Pan Biotech, Aidenbach, Germany) in PBST with 0.02% sodium azide. Sections were then incubated overnight at 4°C with the primary antibodies listed in Table 1. After washing for 3 x 10 minutes in PBST, sections were incubated for 40 minutes at room temperature with the secondary antibodies listed in Table 1. All antibodies were diluted in blocking solution. After washing for 3 x 5 minutes in Tris-buffered saline with Tween 20 (TBST), sections were stained for 20 minutes using the ABC Vectastain Peroxidase Kit (Vector Laboratories; PK-6100) followed by signal development with a Diaminobenzidine Peroxidase Substrate Kit (Vector Laboratories; SK-4100) according to the manufacturer’s instructions. Finally, sections were counterstained with hematoxylin and mounted with Mowiol. Three to 7 (on average 5) representative images were taken at x10 or x20 magnification with an Axioscope 2 equipped with an Axiocam 512 color camera (both from Carl Zeiss Inc). Alternatively, entire sections were scanned using a Pannoramic 250 slide scanner (3DHistech, Budapest, Hungary) at x20 or x40 magnification. Only samples imaged with the same method and the same settings were used for direct comparison. Segmentation of the diaminobenzidine-stained area was done using Ilastik (v1.4.0) software,^61^ followed by quantification in Fiji.

**Table 1:** Antibodies Used for Immunohistochemistry, Western Blotting, and Immunofluorescence

Name	Catalog number	Vendor	Dilution	Diluent	Application
Nrf2 (D1Z9C) XP (R)	12721S	Cell Signaling	1:1000	5% milk in TBST	WB
NF-kappa-B p65 (D14E12) XP (R)	8242S	Cell Signaling	1:1000	5% BSA in TBST	WB
Alpha-tubulin	ab4074	Abcam	1:10000	5% BSA in TBST	WB
GAPDH	5G4	HyTest	1:20000	5% BSA in TBST	WB
Anti mouse-HRP	W402B	Promega	1:5000	5% BSA in TBST	WB
Anti rabbit-HRP	W401B	Promega	1:5000	5% BSA in TBST	WB
CD3	A0452	Agilent Technologies/Dako	1:200	1% BSA in PBS, 0.1% Tween 20	IHC
Ly6G	ab2557	Abcam	1:100	1% BSA in PBS, 0.1% Tween 20	IHC
Biotinylated anti-CD68	MCA1957B	Bio-Rad	1:200 (IHC/IF)	1% BSA in PBS, 0.3% Triton X-100	IHC/IF
Ki67	ab16667	Abcam	1:200	1% BSA in PBS, 0.1% Tween 20	IHC
p-Histone H3 (S10)	9701S	Cell Signaling	1:200	1% BSA in PBS, 0.1% Tween 20	IHC
Clec4f	AF2784	R&D Systems	1:80	1% BSA in PBS, 0.3% Triton X-100	IF
Goat anti-rabbit biotin-conjugated antibodies	111-065-003	Jackson ImmunoResearch	1:500	1% BSA in PBS, 0.1% Tween 20	IHC
Goat anti-mouse biotin-conjugated antibodies	BA-9200	Vector Laboratories	1:500	1% BSA in PBS, 0.1% Tween 20	IHC
Rabbit anti-rat biotin-conjugated antibodies	BA4001	Vector Laboratories	1:100	1% BSA in PBS, 0.1% Tween 20	IHC
Anti-mouse Cy2 (green)	115-225-005	Jackson ImmunoResearch	1:200	1% BSA in PBS, 0.1% Tween 20	IF
Anti-rat Alexa fluor 594 (red)	712-586-150	Jackson ImmunoResearch	1:400	1% BSA in PBS, 0.3% Triton X-100	IF
Anti-goat Alexa Fluor 488 (green)	705-545-147	Jackson ImmunoResearch	1:400	1% BSA in PBS, 0.3% Triton X-100	IF
Anti-alpha-smooth muscle actin	A2547	Sigma	1:400	1% BSA in PBS, 0.3% Triton X-100	IHC

BSA, bovine serum albumin; GAPDH, glyceraldehyde 3-phosphate dehydrogenase; IF, Immunofluorescence; IHC, immunohistochemistry; PBS, phosphate-buffered saline; TBST, Tris-buffered saline with Tween 20; WB, Western blotting.

### Immunofluorescence Staining

Seven-μm unfixed frozen liver sections were washed for 10 minutes in PBS. Sections were fixed in ice-cold acetone for 20 minutes at -20°C, dried for 20 minutes, washed in PBST (PBS with 0.3% Triton X-100), and blocked in 1% BSA in PBST for 1 hour at room temperature. They were incubated overnight at 4°C with the primary antibodies listed in Table 1, followed by washing for 3 x 10 minutes in PBS and incubation for 1 hour at room temperature with the fluorophore-labeled secondary antibodies listed in Table 1. Representative images were taken using an Axioplan 2 microscope (Carl Zeiss Inc) equipped with a monochromatic sCMOS camera (Excelitas Technologies, Waltham, MA).

### Oil Red O Staining

Seven-μm cryosections were washed in PBS for 10 minutes, followed by fixation in 4% PFA for 30 minutes. Slides were briefly washed in 50% isopropanol and stained in a 0.3% solution of oil red O in 60% isopropanol for 10 minutes. They were washed in isopropanol and dH_2_O and counterstained with hematoxylin for 2 minutes. After washing 3 x 10 seconds in dH_2_O, slides were coverslipped using Mowiol 4-88 (Merck, 81381).

### Bile Pigment Staining

Liver samples were fixed in 4% PFA in PBS and embedded in paraffin. Tissue sections (3.5 μm) were deparaffinized and stained with Fouchet’s solution and Van Gieson’s solution and mounted with Mowiol 4-88. Five random images (at x20 magnification) per animal were analyzed. The images were segmented using Ilastik^61^ followed by quantification using the Fiji (ImageJ) software.^62^

### Histology, Histomorphometry, and Image Analysis

PFA-fixed, paraffin-embedded, 3.5-μm sections were used for hematoxylin and eosin, sirius red (picrosirius red stain kit [#ab150681; Abcam, Cambridge, UK]), and Hall’s bilirubin staining. Seven-μm cryosections were used for oil red O staining of lipids. Slides were mounted with either Mowiol (hematoxylin and eosin, sirius red) or Eukitt (oil red O, bilirubin staining). Three to 7 (on average 5) representative images were taken at x20 magnification using an Axioscope 2 microscope equipped with an Axiocam 512 color camera (both from Carl Zeiss Inc). Alternatively, entire sections were scanned using a Pannoramic 250 slide scanner (3DHistech) at x20 or x40 magnification (oil red O). Only samples imaged with the same method at the same settings were used for direct comparison. The necrotic area was determined morphometrically using Fiji software.^62^ Segmentation of the images of sirius red–stained, Hall’s bilirubin–stained, and oil red O–stained sections was done using Ilastik (v1.4.0) software,^61^ followed by quantification in Fiji.

### Oxyblot Analysis

Fresh pieces of liver tissue (∼20 mg) were homogenized in 500 μL of T-PER tissue protein extraction reagent (Thermo Fisher Scientific; 78510) containing cOmplete EDTA-free Protease Inhibitor Cocktail (Roche; 40694200), PhosSTOP Phosphatase Inhibitor Cocktail (Roche; 04906837001), and 1% β-mercaptoethanol. Oxidized proteins were detected using the OxyBlot Protein Oxidation Detection Kit (S7150, Merck-Millipore, Burlingame, MA), according to the manufacturer’s manual.

### Serum Analysis

Blood was taken from the inferior vena cava before perfusion. After coagulation, samples were centrifuged at 7000 x *g* for 5 minutes. The supernatant was removed and snap-frozen in liquid nitrogen and stored at -80°C. For analysis, serum samples were thawed and brought to room temperature before use. Samples from intact liver were diluted 1:2 in 0.9% saline for ALT measurements. 1:20/1:40 dilutions were used for ALT measurement in the serum of CCl_4_-treated mice. Higher dilutions were used when the measured level exceeded the linear range. Diluted samples (32 μL) were loaded onto the red application zone of the Reflotron GPT (ALT) (Roche; 10745138) test strips and measured by the Roche Reflotron Plus system.

### Bile Acid Measurement

Frozen liver tissue was homogenized in methanol/water (50/50, vol/vol) using bead-based homogenization at a concentration of 0.05 mg/μL. Liver homogenate representing 2 mg (wet weight) was used for bile acid quantification by liquid chromatography–tandem mass spectrometry as recently described in detail.^63^

### RNA Isolation and RT-qPCR

Total cellular RNA from cultured cells was isolated using the Total RNA Mini Kit (IBI Scientific, Dubuque, IA; IB47303) according to the manufacturer’s instructions, followed by cDNA synthesis using an iScript cDNA Synthesis Kit (Bio-Rad Laboratories; 64274497 and 64211314). qPCR was performed in a 10-μL reaction mixture containing 5 μL of Light Cycler 480 Sybr Green PCR Master Mix (Roche; 4887352001) and 0.6 mM of each primer. Analysis was performed using the ΔΔCt method with glyceraldehyde-3-phosphate dehydrogenase (*Gapdh*) as a reference gene. Primers used for qPCR are listed in Table 2.

**Table 2:** Primer Sequences Used for RT-qPCR

Primer name	Direction	Primer sequence
*Gclc*	Forward	CGTAGCCTCGGTAAAATGGA
	Reverse	AACAAGAAACATCCGGCATC
*Nqo1*	Forward	GTCTGCAGCTTCCAGCTTCT
	Reverse	CTGGCCCATTCAGAGAAGAC
*Nrf2*	Forward	ACAAGCAGCTGGCTGATACTACC
	Reverse	CCAGCGAGGAGATCGATGAGTA
*p65*	Forward	TTCCTGGCGAGAGAAGCAC
	Reverse	AAGCTATGGATACTGCGGTCT
*Saa3*	Forward	TGGGAGTTGACAGCCAAAGA
	Reverse	TAGGCTCGCCACATGTCTCT
*Gapdh*	Forward	CACCACCCTGTTGCTGTAGCCGTAT
	Reverse	TCGTGGATCTGACGTGCCGCCTG
*Col1a1*	Forward	TGTTCAGCTTTGACCTCCGGCT
	Reverse	TCTCCCTTGGGTCCCTCGACT
*Timp1*	Forward	GCCCCCTTTGCATCTCTGGCAT
	Reverse	TGCGGCATTTCCCACAGCCT
*Cyp7a1*	Forward	GGGATTGCTGTGGTAGTGAGC
	Reverse	GGTATGGAATCAACCCGTTGTC
*Cyp27a1*	Forward	CCAGGCACAGGAGAGTACG
	Reverse	GGGCAAGTGCAGCACATAG
*Mrp2*	Forward	CCTGGAAATCACGATGGACGA
	Reverse	CAGGAGCCAAGTGCATAGGT
*Mrp3*	Forward	GCAGAGACAGGCAATGTGAA
	Reverse	GAAAGCTGACAGCATGACCA
*Abcg2*	Forward	TGGACTCAAGCACAGCGAAT
	Reverse	ATCCGCAGGGTTGTTGTAGG
*Bsep*	Forward	TCTGTAGGGCCTTCTCTGCT
	Reverse	CCATTCCATGGAGCAATGCG

RT-qPCR, quantitative reverse transcription polymerase chain reaction.

### Preparation of Protein Lysates and Western Blot Analysis

Hepatocyte or NPC pellets were lysed in T-PER tissue protein extraction reagent (Thermo Fisher Scientific; 78510) containing cOmplete EDTA-free Protease Inhibitor Cocktail (Roche; 40694200) and PhosSTOP Phosphatase Inhibitor Cocktail (Roche; 04906837001). Lysates were sonicated for 5 x 15 seconds at 30% amplitude and 50% cycle (IKA U200S control; IKA Werke, Staufen, Germany), followed by centrifugation (17000 x *g*, 30 minutes, 4°C) in a minicentrifuge. The supernatant was transferred to a new tube and stored at -80°C. The protein concentration was determined using the Pierce BCA Protein assay kit (Thermo Fisher Scientific; 23225).

Proteins were separated in 4%–20% gradient sodium dodecyl sulfate-polyacrylamide gel electrophoresis and transferred onto Immobilon-P PVDF membranes (Merck-Millipore; IPVH00010). Unspecific binding sites were blocked by incubation of the membrane in 5% BSA in TBST (20 mM Tris, 150 mM NaCl, 0.1% Tween 20) at room temperature for 1 hour before incubating with primary antibody solutions overnight at 4°C. Primary and secondary antibodies used for Western blot experiments are listed in Table 1. Membranes were washed for 3 x 5 minutes in TBST and incubated with the secondary antibody for 1 hour at room temperature. Finally, membranes were treated with WesternBright ECL HRP substrate (Advansta, San Jose, CA; K-12045-D50) or WesternBright Sirius HRP substrate (Advansta; K-12043-D10), and chemiluminescence signals were detected using a Fusion Solo S developer (Vilber Lourmat, Eberhardzell, Germany).

### RNA Sequencing

Total RNA from freshly isolated hepatocytes of control, *Nrf2*, *p65* single- and *Nrf2:p65* DKO mice, 12 days after AAV8Cre injection, was isolated as described previously. All mice were injected and sacrificed in the morning. RNA quality was assessed using a TapeStation (Agilent Technologies, Santa Clara, CA). RNA samples with an RQN ≥8 were subjected to the RNA sequencing protocol via poly-A enrichment, True-Seq library preparation, and single-end 100 bp sequencing on a Novaseq 6000 instrument (Illumina, San Diego, CA).

### RNA-Seq Data Analysis

Alignment of reads was performed using Salmon software, version 1.10.3^64^ using the following parameters: quant -l SF –fldMean 170 -fldSD 50 --seqBias --validateMappings, and reference genome version GRCm39 from the GENCODE project with gene model annotation version M33. For differential expression analysis, the DESeq2 software, version 1.42.1^65^ with default parameters, and Benjamini-Hochberg multiple testing correction were used. Before differential expression analysis, weakly expressed genes with less than 10 estimated counts were filtered out. Functional analysis was performed based on the significantly regulated genes (FDR ≤0.05 and a log2 fold change greater/lower 1/-1 respectively) using R (version 4.1.1) and clusterProfiler package (version 4.4.4).^66^ Enrichment was determined in Gene Ontology (Biological Processes, Molecular Functions, and Cellular Components) gene sets at FDR <0.05.

### ChIP-seq Data Analysis

The data of previously published ChIP-seq experiments for Nrf2 and p65 in the mouse liver were taken from the Gene Expression Omnibus database (https://www.ncbi.nlm.nih.gov/geo/). Fastq files corresponding to the Nrf2 (GSM2971838 input, GSM2971839 immunoprecipitation) and p65 samples (GSM3301835 and GSM3301836 input, GSM3301805 and GSM3301806 immunoprecipitation) were downloaded and aligned against the mouse genome GRCm39 version M33 using Bowtie2 v2.5.3^67^ in a --local mode. After filtering out duplicated reads using Sambamba v1.0.0 software,^68^ peaks were called using MACS2 v2.2.9.1.^69^ Only strong peaks (fold enrichment relative to the input >10 and -log10(FDR) ≥20) within -15000 kb to +500 kb from the transcription start site were considered. Further analysis of peak data was performed using R and ChIPseeker v1.38.0.^70^

### Flow Cytometry

NPCs were isolated as outlined previously and resuspended in 1 mL of fluorescence-activated cell sorting (FACS) buffer (1 x PBS, 5 mM EDTA, 0.2% BSA). Antibodies and staining dyes used are listed in Table 3. One-third of the obtained cell suspension was taken to stain myeloid cells. After incubating cells in 50 μL of Fc receptor blocking mAb (1:200; clone 2.4G2) for 5 minutes and washing in 150 μL FACS buffer, cells were incubated for 15 minutes in 50 μL of FACS buffer containing the myeloid panel antibody cocktail. After washing in 200 μL FACS buffer and 200 μL PBS, cells were fixed with 1.48% formaldehyde in PBS for 10 minutes, washed, and resuspended in 250 μL FACS buffer and filtered through the 60-μm cell strainer before the signal was acquired.

**Table 3:** Antibodies Used for Flow Cytometry

Antibody	Channel	Catalog number	Supplier	Dilution
CD16/CD32	Fc block	553142	BD Biosciences	1:200
CD11c	BV605	117333	BioLegend	1:400
CD45	BV785	103149	BioLegend	1:1000
eFluor 780	APC-Cy7	65-0865-14	Invitrogen	1:2000
NK1.1	PE	12-5941-82	eBioscience	1:300
CD11b	BV510	101263	BioLegend	1:400
CD19	PE	12-0193-82	eBioscience	1:500
CD3	PE	12-0031-82	eBioscience	1:300
CD64	BV421	139309	BioLegend	1:200
F4/80	APC	123116	BioLegend	1:200
I-A/I-E	BV650	107641	BioLegend	1:4000
Ly6C	PE-Cy7	128018	BioLegend	1:5000
Ly6G	Alexa Fluor 700	127622	BioLegend	1:400
PDCA-1	FITC	127007	BioLegend	1:400
Siglec-F	PE	552126	BD Biosciences	1:300
Streptavidin	BV711	563262	BD Biosciences	1:500
XCR-1	PerCP-Cy5.5	148208	BioLegend	1:300

The cell suspension was acquired on an LSRFortessa Analyzer (BD Biosciences, Franklin Lakes, NJ). Flow cytometry data were analyzed using FlowJo software,^71^ and statistical analysis was performed using Prism9 software (GraphPad Software Inc, San Diego, CA).

### Treatment of Primary Hepatocytes with Growth Factors

Freshly isolated hepatocytes were counted and plated at a density of 25,000 cells/cm^2^ onto collagen-coated 6-well plates in 3 mL/well of Williams E medium (12551032, Gibco/Thermo Fisher Scientific) supplemented with GlutaMax (35050061, Gibco/Thermo Fisher Scientific) and gentamycin (50 μg/mL). Three hours after plating, cells were washed with PBS and the same medium, containing 20 ng/mL of murine hepatocyte growth factor (315-23, PeproTech, Cranbury, NJ) or 20 ng/mL murine epidermal growth factor (E4127- 5X.1MG, Sigma-Aldrich), was added. On the following day, the medium was replaced by fresh medium containing growth factors and in addition 100 μM BrdU. Cells were incubated for 24 hours at 5% CO_2_/37°C. The protocol for the detection of BrdU-positive cells is described later.

### Cell Culture

AML12 cells were cultured and maintained in DMEM:F12 (1:1) GlutaMax (Thermo Fisher Scientific; 10565018) supplemented with 1% P/S and 10% FBS at 37°C and 5% CO_2_ in tissue culture flasks. Cells were regularly tested for mycoplasma contamination using the PCR Mycoplasma Test Kit I/C (Vitaris AG, Baar, Switzerland; PK-CA91-1096).

### CRISPR/Cas9-Mediated KO in AML12 Cells

The following oligonucleotides, used as guide RNAs, were annealed, and cloned into a pSpCas9(BB)-2A-Puro (PX459) V2.0 vector (Addgene; 62988) backbone using the *BbsI* restriction site:

p65 forward: 5'-CAC CGC GAT TCC GCT ATA AAT GCG-3'

p65 reverse: 5'-AAA CCG CAT TTA TAG CGG AAT CGC-3'

Nrf2 forward: 5'-CAC CGT GAC TTT AGT CAG CGA CAG A-3'

Nrf2 reverse: 5'-AAA CTC TGT CGC TGA CTA AAG TCA C-3'

AML12 cells were seeded into 6-well plates and transfected with the gRNA expression vectors using Lipofectamine 2000 (Invitrogen, Waltham, MA). After overnight incubation, cells were trypsinized and seeded into 10 cm^2^ dishes in the same medium containing 1 μg/mL puromycin (Sigma-Aldrich; P8833) for 24 hours. The medium was replaced by fresh medium without puromycin, and cells were left to recover for 48 hours before sorting single cells into 96-well plates for clonal expansion and screening. KO cells were identified by Sanger sequencing, RT-qPCR, and Western blot analysis.

### Analysis of the Proliferation Rate of AML12 Cell Lines

Twenty thousand cells were seeded per well into 24-well plates in triplicate in DMEM:F12 (1:1) GlutaMax (Thermo Fisher Scientific; 10565018), supplemented with 1% P/S and 10% FBS at 37°C and 5% CO_2_. The next day, the medium was replaced with the same medium, but containing 100 μM of BrdU, incubated for 1.5 hour, and the cells were stained as described later.

### siRNA-Mediated Knockdown in AML12 Cells and BrdU Assay

Double-stranded siRNAs were synthesized by Microsynth (Balgach, Switzerland). The following sequences were used:

siNrf2_1 sense: 5’-GCA AGA AGC CAG AUA CAA A-dTdT-3’

siNrf2_1 antisense: 3’-dTdT-UUU GUA UCU GGC UUC UUG C-5’

sip65_1 sense: 5'-GAA GAG UCC UUU CAA UGG A-dTdT-3'

sip65_1 antisense: 3'-dTdT-UCC AUU GAA AGG ACU CUU C-5'

Scrambled siRNA sense: 5'-ACG UGA CAC GUU CGG AGA AUU dTdT-3'

Scrambled siRNA antisense: 3’-dTdT UGC ACU GUG CAA GCC UCU UAA-5’.

Six picomole siRNA was prediluted in 50 μL of Opti-MEM-I–reduced serum medium (Thermo Fisher Scientific; 31985-047) and mixed with prediluted Lipofectamine RNAi MAX (Invitrogen; 56532) (1 μL of lipofectamine in 50 μL of Opti-MEM). After 5 minutes, the siRNA:lipofectamine suspension was pipetted into 1 well of a 24-well plate, and 20,000 cells in 0.4 mL of DMEM:F12, 10% FBS, without antibiotics were added. Seventy-two hours later, cells were incubated with the same medium containing 100 μM BrdU, incubated for another 1.5 hours, and stained as described next.

### Staining of BrdU-Labeled Cells

After incubation with BrdU, cells were washed in PBS, fixed in 4% formaldehyde for 30 minutes at room temperature, and incubated with 2 N HCl, 0.1% Triton X-100 for 30 minutes at room temperature. After neutralization in 0.1 M sodium tetraborate pH 8.5 for 5 minutes, cells were washed in PBS, blocked in 1% BSA in PBST, and incubated overnight at 4°C with anti-BrdU antibody (1202693, Roche; 1:200 diluted in blocking buffer). The next day, they were washed 3 times in PBST and incubated for 1 hour with goat anti-mouse-Cy2 secondary antibody (115-225-005, Jackson ImmunoResearch; 1:100 diluted), and Hoechst 33342 (Thermo Fisher Scientific, H3570; 1:1000 diluted) in blocking buffer, washed in PBS and mounted with Mowiol:DABCO. From each well, 8 representative images were taken using an Axiovert fluorescence microscope (Carl Zeiss Inc) equipped with a monochromatic camera (Axiocam 506 mono; Carl Zeiss Inc), and the number of BrdU-positive nuclei and the total number of nuclei were determined by image segmentation using Ilastik (v1.4.0) software,^61^ followed by quantification in Fiji.

### Statistical Analysis

Statistical analysis was performed with the Prism10 software (GraphPad Software). Quantitative data are shown as bar graphs with mean and standard error of the mean. Unless otherwise stated, significance was calculated using the Mann-Whitney *U* or 2-way analysis of variance tests. ∗*P* ≤ .05, ∗∗*P* ≤ .01, ∗∗∗*P* ≤ .001, ∗∗∗∗*P* ≤ .0001.
